# Mechanisms and therapeutic advances of gut metabolites in the regulation of neuroimmune inflammatory diseases

**DOI:** 10.3389/fimmu.2026.1795042

**Published:** 2026-06-02

**Authors:** Xiaodan Shen, Caiji Zheng, Renyong Lin, Juan Wang, Zheng Chen

**Affiliations:** 1 Department of Pharmacy, Shenzhen Mental Health Center/Shenzhen Kangning Hospital, Shenzhen, China; 2 Department of Medical Affairs, Shenzhen Longhua District Central Hospital/Shenzhen University Affiliated Longhua Hospital, Shenzhen, China

**Keywords:** gut metabolites, gut-immune-brain axis, immunomodulation, neuroimmune inflammatory diseases, precision medicine

## Abstract

Gut-derived metabolites function as critical signaling intermediaries that translate environmental cues into central nervous system (CNS) responses, playing an indispensable role in the pathogenesis and trajectory of neuroimmune inflammatory disorders. Key metabolites, including short-chain fatty acids (SCFAs) and bile acids, either traverse the blood-brain barrier directly or orchestrate immune modulation peripherally, thereby fine-tuning the dynamic crosstalk between systemic immunity and neural homeostasis. SCFAs exert potent anti-inflammatory effects by promoting regulatory T-cell (Treg) differentiation through activation of G protein-coupled receptors (GPCRs) on immune cells and inhibition of histone deacetylases (HDACs). Within the CNS, they further confer neuroprotection by suppressing the pro-inflammatory activation of microglia and astrocytes. In contrast, bile acids display a context-dependent, “double-edged sword” effect: while certain subtypes activate the anti-inflammatory TGR5 receptor, neurotoxic metabolites (e.g., taurolithocholic acid) can accumulate and directly provoke pro-inflammatory polarization of microglia, thereby fueling neuroinflammation. Dysbiosis of the gut microbiota and consequent metabolite profile alterations are strongly implicated in neuroimmune inflammatory diseases—such as multiple sclerosis (MS), Alzheimer’s disease (AD), and neuromyelitis optica spectrum disorders (NMOSD) —which are characterized by both a distinct metabolite imbalance and a pervasive pro-inflammatory immune milieu. Building on this framework, novel therapeutic strategies targeting the “gut-immune-brain axis” are evolving along two complementary avenues: (1) Immune-centric approaches that directly modulate neuroimmune pathways (e.g., by tempering microglial activation or expanding Treg populations); and (2) Microbiota-centric interventions that employ specific probiotics, prebiotics, or metabolite supplements to restore gut ecological balance, systemically recalibrate immunity, and mitigate neuroinflammation. Future research must prioritize elucidating the precise molecular dialogues between metabolites and immune cell subsets, conducting large-scale clinical validation, and advancing personalized, precision-medicine strategies. Such efforts will solidify a novel systemic perspective and strategic paradigm for preventing and treating neuroimmune inflammatory diseases.

## 1 Introduction

**Figure 1:**
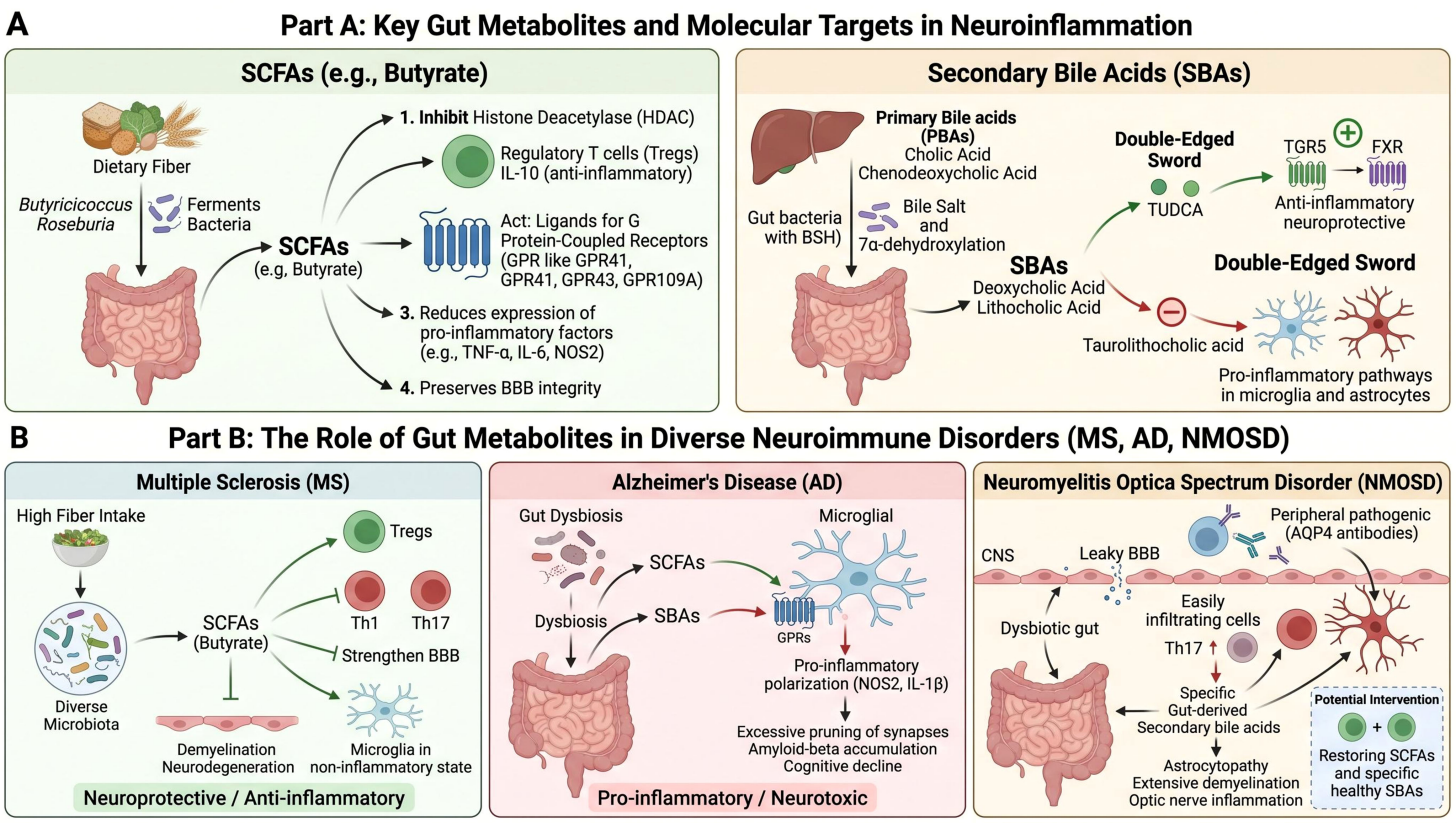
Molecular mechanisms and disease-specific roles of gut-derived metabolites in neuroinflammation. **(A)** The major molecular targets and mechanisms of two key classes of gut-derived metabolites, short-chain fatty acids (SCFAs) and secondary bile acids (SBAs), in neuroinflammation. Dietary fibers are fermented by gut bacteria, such as Butyricicoccus and Roseburia, to generate SCFAs, including butyrate. SCFAs exert anti-inflammatory and neuroprotective effects by inhibiting histone deacetylases (HDACs), activating G protein-coupled receptors such as GPR41, GPR43, and GPR109A, promoting regulatory T cell (Treg) responses and IL-10 production, reducing the expression of pro-inflammatory mediators including TNF-α, IL-6, and NOS2, and preserving blood–brain barrier integrity. Primary bile acids are converted by gut bacteria through bile salt hydrolase activity and 7α-dehydroxylation into SBAs. SBAs may exert context-dependent effects: TUDCA can activate receptors such as TGR5 and FXR to mediate anti-inflammatory and neuroprotective responses, whereas metabolites such as taurolithocholic acid may enhance pro-inflammatory pathways in microglia and astrocytes. **(B)** Disease-specific roles of gut-derived metabolites in multiple sclerosis (MS), Alzheimer’s disease (AD), and neuromyelitis optica spectrum disorder (NMOSD). In MS, high-fiber intake and increased SCFA production promote Treg differentiation, suppress Th1/Th17 responses, strengthen the blood–brain barrier, and reduce demyelination and neurodegeneration. In AD, gut dysbiosis and altered SCFA/SBA signaling may drive microglial pro-inflammatory polarization, excessive synaptic pruning, amyloid-β accumulation, and cognitive decline. In NMOSD, dysbiotic gut microbiota and disease-associated secondary bile acid profiles may increase blood–brain barrier permeability, facilitate the entry of peripheral pathogenic immune cells and AQP4 antibodies into the CNS, and contribute to astrocytopathy, extensive demyelination, and optic nerve inflammation. Restoring SCFA levels and modulating specific beneficial SBAs may represent potential therapeutic strategies.

The pathological core of neuro-immune inflammatory disorders lies in aberrant immune activation and dysregulation, characterized not only by the recruitment and infiltration of specific immune cells into the central nervous system (CNS) but also by the excessive release of a series of pro-inflammatory mediators, such as reactive oxygen species, prostaglandins, cytokines, and chemokines (1). This persistent inflammatory microenvironment disrupts the structural and functional integrity of the blood-brain barrier (BBB), facilitating the invasion of peripheral immune cells into the CNS parenchyma and thereby amplifying and sustaining inflammatory damage within the brain, forming a vicious cycle (2). Substantial evidence indicates that such neuro-immune inflammatory responses are a key common mechanism driving the onset and progression of various neuroimmune inflammatory diseases, including multiple sclerosis (MS), Alzheimer’s disease (AD), and Parkinson’s disease (PD) (3–6) (**Figure 1**).

Traditionally, the CNS was regarded as an “immune-privileged” organ (7). However, this notion has been thoroughly revised by modern neuroimmunology (8). It is now recognized that the CNS harbors a highly complex and dynamically regulated innate immune ecosystem, comprising resident microglia and astrocytes, border-associated macrophages, and a certain number of tissue-resident adaptive immune cells (such as T and B lymphocytes). This system plays an indispensable role in maintaining neural development, synaptic plasticity, tissue homeostasis, and higher cognitive functions (9). Meanwhile, the gut, as the largest symbiotic microbial habitat and a peripheral immune organ in the human body, has its vast microbial community and their derived metabolites proven to be key environmental determinants shaping the host’s lifelong immune phenotype and function (10). In early life, maternal microbiota and their metabolites (such as short-chain fatty acids) can cross the placenta and influence the primary programming of the fetal immune system (11); after birth, diet and environment rapidly shape the neonatal gut microbiota, whose metabolic product repertoire becomes the core biochemical instruction set driving the maturation and functional differentiation of the immune system (12).

Particularly important is the close bidirectional communication between the gut microbiota and the nervous system via the “gut-microbiota-brain axis,” profoundly influencing neural development and function. Studies using germ-free mouse models have revealed that the absence of gut microbiota impairs several fundamental processes, including neuronal development, myelination, and neural regeneration, and leads to significant defects in microglia in terms of morphology, density, and function. Subsequent mechanistic studies have indicated that bacterial metabolites, especially short-chain fatty acids (SCFAs), are the key mediators of these effects (13). Through these specific molecular pathways, gut metabolites can directly or indirectly act on resident cells within the CNS, not only regulating the functional state of astrocytes but also potentially initiating or alleviating the pathological cascade of neuro-immune abnormalities (14) (**Figure 1A**).

Breakthrough findings in recent years have further demonstrated that specific gut metabolites can cross biological barriers (such as the blood-brain barrier) or actively intervene in the neuroinflammatory process by precisely regulating the state of peripheral and central immune cells. For example, SCFAs (such as butyrate) act as histone deacetylase (HDAC) inhibitors and ligands for G protein-coupled receptors (such as GPR41/43, GPR109A), not only inhibiting the pro-inflammatory polarization of microglia and promoting the differentiation and function of regulatory T cells (Tregs) but also demonstrating direct neuroprotective potential and the ability to repair the blood-brain barrier (15, 16). The dynamic balance of the bile acid spectrum presents a complex “double-edged sword” effect: on one hand, it exerts anti-inflammatory and neuroprotective effects through activation of the TGR5 receptor (17); on the other hand, gut dysbiosis leading to an abnormal proportion of microbes with bile salt hydrolase activity may produce neurotoxic secondary bile acids, such as taurolithocholic acid, which directly induce microglia to adopt a pro-inflammatory phenotype (18). In addition, gut microbiota can synthesize or influence the precursors of various neuroactive substances (such as dopamine, serotonin, γ-aminobutyric acid), which can affect the brain’s neurotransmitter balance, mood, and cognitive function through the circulatory system or the enteric nervous system (ENS) (19). Other metabolites, such as polyamines (spermine, spermidine, etc.), can also be recognized by receptors on intestinal epithelial and immune cells, participating in neuro-immune regulation and stress responses. Although these mechanisms are becoming increasingly clear, systematic research on how specific gut metabolite profiles dynamically influence the progression of particular neuro-immune inflammatory disorders remains relatively scarce, and the translational value of these metabolites as disease classification biomarkers or intervention targets are needed to explore.

Therefore, systematically elucidating how gut metabolites act as core biochemical messengers, reshaping local and systemic immune homeostasis, and thereby influencing brain health and disease status through this bridge, is of key significance for fundamentally understanding the pathogenesis of neuropsychiatric disorders and developing novel intervention strategies. What distinguishes this review from previous work is its metabolite-centered perspective. Rather than focusing only on the correlation between gut microbiota dysbiosis and neurological diseases, we highlight gut-derived metabolites as active biochemical messengers that link the intestinal ecosystem to peripheral immune responses and central neuroinflammation.

## 2 Literature search strategy

Databases searched (PubMed, Web of Science up to 2025). Search terms (e.g., “gut-derived metabolites,” “short-chain fatty acids,” “bile acids,” “neuroinflammation,” “multiple sclerosis,” “gut-immune-brain axis”). Inclusion criteria (peer-reviewed original articles and reviews in English; studies on humans or animal models addressing inflammatory neuroimmune diseases). Exclusion criteria (non-English articles, conference abstracts without full text, studies solely on non-inflammatory neurological disorders). Explicitly discussed potential selection bias in the revised Discussion section. We acknowledge that despite our systematic approach, some relevant studies may have been missed, and we have noted this as a limitation.

## 3 Core pathways of gut metabolite-mediated gut-brain axis communication

The gut microbiota constructs a dynamic and adjustable “gut-brain axis” communication network through its metabolites, playing a crucial regulatory role in maintaining central nervous system homeostasis and influencing various disease processes (20). These metabolites primarily exert their biological effects through two core mechanisms: first, as signaling molecules capable of crossing biological barriers like the blood-brain barrier to directly act on specific receptors on the surface of neurons or glial cells (such as microglia); second, by modulating the state of peripheral and central immune systems to indirectly influence the neuroinflammatory microenvironment (21). Therefore, gut metabolites serve not only as energy substrates or structural components but also play multiple roles as epigenetic regulators, G protein-coupled receptor (GPCR) agonists, and immune regulatory hubs, collectively forming a complex metabolic-immune-neural signaling network (21, 22).

## 4 Gut metabolites: microscopic architects of immune homeostasis

The early stages of life represent a critical window for establishing a lifelong “dialogue” between the gut microbiota and the host immune system (23). Maternal microbiota and their metabolites, such as SCFAs and secondary bile acids, can traverse the placental barrier during gestation to influence the development and environmental shaping of fetal immune precursor cells (11). Postnatally, factors including diet (particularly breastfeeding), environment, and early antibiotic exposure rapidly sculpt the neonatal microbial ecosystem. The metabolites derived from this microbiota serve as core signals that drive the maturation and functional programming of the immune system (24). Studies using germ-free (GF) mouse models have demonstrated reductions in various immune cells (e.g., macrophages, T/B cells), functional defects, and incomplete development of mucosal lymphoid structures, all of which underscore the foundational role of symbiotic microbiota and their metabolites in immune system construction (25, 26). Consequently, the library of metabolites derived from the gut microbiota functions as a biochemical bridge connecting the external environment with the host’s internal immune development.

Within the mature gut mucosal immune network, metabolites act as precise regulators, maintaining a dynamic equilibrium. For instance, SCFAs—the primary products of dietary fiber fermentation—bind to G protein-coupled receptors (GPR41, GPR43, GPR109A) on immune cells, thereby inhibiting pro-inflammatory pathways such as NF-κB (27). Additionally, SCFAs function as HDAC inhibitors (28), epigenetically promoting the differentiation of regulatory T cells (Tregs) and the production of anti-inflammatory factors like IL-10, thus suppressing excessive inflammation (29). Conversely, specific microbial communities, such as segmented filamentous bacteria, can drive the polarization of T helper 17 (Th17) cells, leading to the generation of IL-17 to bolster mucosal barrier defense (30, 31) (**Figure 1A**). Beyond SCFAs, microbial-associated molecular patterns (MAMPs), such as lipopolysaccharide (LPS), activate innate immune responses via Toll-like receptors (e.g., TLR4) (32), while probiotic surface components can induce Treg responses through TLR2 (33). This illustrates the precision with which microbiota metabolites or surface components can directly modulate immunity.

## 5 Gut metabolites and neurotransmitter dysregulation in neuroimmune inflammatory diseases

A spectrum of gut-derived metabolites, including neurotransmitters such as 5-hydroxytryptamine (5-HT), dopamine, and γ-aminobutyric acid (GABA), are implicated in the pathophysiology of neuropsychiatric disorders like depression (22). These metabolites exert multifaceted influences on central neurotransmitter systems through both direct and indirect mechanisms (34). For instance, short-chain fatty acids (SCFAs) like butyrate and propionate, produced by microbial fermentation of dietary fiber, can traverse the blood-brain barrier. Butyrate promotes neurogenesis via histone acetylation, thereby indirectly modulating neurotransmitter synthesis and release (35), whereas propionate may alter synaptic morphology and connectivity, impacting neurotransmission efficiency. Furthermore, the gut microbiota orchestrates the tryptophan metabolism pathway, generating critical neuromodulators including 5-HT and kynurenine (36). While 5-HT serves as a pivotal neurotransmitter regulating mood and sleep, dysregulation of the kynurenine pathway is closely linked to neuroinflammation and imbalances in key neurotransmitters such as dopamine and glutamate.

Beyond direct metabolic interactions, gut metabolites disrupt neurotransmitter homeostasis via immune modulation. SCFAs promote the differentiation of regulatory T cells (Tregs) and suppress the release of pro-inflammatory cytokines such as IL-6 and TNF-α. These cytokines can directly impair neuronal function, leading to aberrant neurotransmitter synthesis, release, or reuptake—a phenomenon observed in neuroimmune inflammatory diseases as suppressed dopaminergic neuron activity and reduced dopamine levels. Concurrently, gut metabolite dysbiosis can compromise blood-brain barrier integrity, facilitating the infiltration of peripheral immune cells and harmful substances like lipopolysaccharide (LPS) into the central nervous system (37). This cascade activates microglia, exacerbates neuroinflammation, and further disrupts neurotransmitter equilibrium. Notably, the gut-brain axis features bidirectional communication: neurotransmitters (e.g., acetylcholine) can modulate gut microbiota composition and metabolism via the vagus nerve, while stress-induced activation of the hypothalamic-pituitary-adrenal (HPA) axis releases glucocorticoids that alter the gut metabolome, which in turn provides feedback to regulate neurotransmitter release, forming a complex regulatory circuit. However, within the specific context of neuroimmune inflammatory diseases, SCFAs and secondary bile acids have been identified as two key effector molecules (37). Based on this, the following sections will focus on elucidating the primary molecular mechanisms through which SCFAs and secondary bile acids mediate the onset and progression of these diseases (**Figure 1B**).

## 6 Gut metabolites regulating neuroimmune inflammatory diseases

Neuroimmune inflammatory diseases are a group of disorders characterized by immune cell or immune molecule-mediated attacks on the nervous system, leading to pathological changes such as varying degrees of neuronal or axonal damage, demyelination, and disruption of neuromuscular junctions. These diseases can occur in the CNS, the peripheral nervous system (PNS), and at the neuromuscular junction. In the CNS, representative diseases include MS, NMOSD, and AE. In the PNS, Guillain-Barré syndrome (GBS) is a key example, while myasthenia gravis (MG) represents neuromuscular junction pathology. Among the numerous metabolites involved, SCFAs, such as acetate, propionate, and butyrate, bile acids (BAs, such as ursodeoxycholic acid and tauro-beta-muricholic acid), neurotransmitter precursors (such as tryptophan derivatives), and trimethylamine N-oxide (TMAO) have emerged as current research foci due to their well-defined biological activities and abundance in regulating these diseases (**Figure 1B**).

### 6.1 SCFAs

SCFAs are organic compounds with a low number of carbon atoms (2-5), primarily produced by gut microbiota through the anaerobic fermentation of dietary fiber (38). The major SCFAs include acetate (C2), propionate (C3), butyrate (C4), and valerate (C5) (38). In the colon and feces, their molar ratio is typically approximately 60:20:20 for acetate, propionate, and butyrate, respectively. SCFAs act as crucial mediators in the gut-brain axis, being absorbed into systemic circulation and exerting multifaceted effects at cellular and molecular levels (39). In contrast, medium-chain fatty acids like caproate (C6), which can induce Th1 and Th17 lymphocyte differentiation (40), are directly derived from diet or hepatic peroxisomal β-oxidation of long-chain fatty acids; their mechanisms fall outside the scope of this review (**Figure 1A**).

As major metabolites of commensal bacteria, SCFAs can cross the blood-brain barrier (BBB) and directly modulate immune cells within the CNS. Butyrate, functioning as a HDAC inhibitor, demonstrates potential in improving cognition and memory in models of neurodegenerative diseases such as AD and Parkinson’s disease (PD). This is mechanistically linked to enhanced cerebral histone acetylation and suppressed expression of pro-inflammatory genes (e.g., TNF-α, IL-6, NOS2) in microglia (**Figure 1B**). In ischemic stroke models, butyrate and acetate inhibit neuronal apoptosis and promote IL-22-dependent angiogenesis and BBB repair via activation of the GPR41/PI3K/Akt pathway. Acetate also mitigates lipopolysaccharide (LPS)-induced production of pro-inflammatory cytokines (IL-1β, IL-6, TNF-α) in microglia and astrocytes, involving the inhibition of NF-κB and MAPK p38 signaling. Notably, aberrant microglial morphology and function observed in GF mice can be partially rescued by SCFAs supplementation or expression of their cognate G protein-coupled receptors (GPCRs), such as FFAR2 (GPR43), underscoring the foundational role of SCFAs in neuroimmune cell development and function.

Regarding BBB integrity, while a small fraction of intravenously infused acetate crosses the BBB to stimulate hypothalamic neurons and promote satiety (41), colonization of GF mice with butyrate-producing C. tyrobutyricum and acetate/propionate-producing B. theaiotaomicron, or oral sodium butyrate administration, reduces BBB permeability. This effect correlates with increased occludin expression in the hypothalamus and frontal cortex (42). Intraperitoneal and intravenous sodium butyrate injections similarly inhibit BBB disruption while promoting neurogenesis and angiogenesis (43, 44). Collectively, these findings indicate that SCFAs exert bioactive effects in diverse brain regions, influencing neurogenesis, host barrier function, and behavior. Clinically, patients in the acute phase of neuromyelitis optica spectrum disorder (NMOSD) exhibit gut dysbiosis and acetate deficiency. Fecal microbiota transplantation from NMOSD patients to GF mice, or direct acetate supplementation, elevates serum pro-inflammatory cytokines (IL-6, IL-17A, IL-23), increases Th17 cell proportion, and decreases Treg cells, identifying acetate as a potential diagnostic and therapeutic target for NMOSD (45) (**Figure 1B**).

Research into the precise immunomodulatory mechanisms of SCFAs in neuroimmune inflammatory diseases reveals that butyrate primarily signals through GPCRs, including GPR41, GPR43, and GPR109α, to trigger intracellular pathways associated with immune regulation (46, 47). GPR43 is implicated in the development and function of brain immune cells, the most abundant being microglia (48). SCFA supplementation normalizes microglial morphology in GF mice, whereas GPR43-deficient mice exhibit microglial abnormalities similar to GF mice. However, since GPR43 is not expressed in microglia, its role requires further elucidation. In contrast, GPR109α is selectively expressed in microglia and is significantly upregulated in the AD brain. GPR109α-deficient AD mouse models show impaired microglial response to amyloid-β deposits, leading to exacerbated neuronal loss (49). Conversely, pharmacological activation of GPR109α with the FDA-approved niacin formulation Niaspan reduces amyloid pathology and attenuates neuronal loss in AD models, indicating that GPR109α is essential for the neuroprotective function of microglia against amyloid pathology.

Butyrate also promotes myelination and repair, validated in multiple sclerosis (MS) models through its HDAC inhibitory activity (50). It inhibits demyelination and enhances remyelination by promoting oligodendrocyte differentiation (51). In experimental autoimmune encephalomyelitis (EAE), a classic mouse model of MS, fecal microbiota transplantation from inulin-treated (butyrate-enriched) donor mice significantly ameliorates disease in recipient EAE mice. This improvement is accompanied by reduced inflammatory cell infiltration and demyelination in the CNS, correlated with decreased proportions and numbers of Th17 cells in the brain (52).

The regulatory role of gut metabolites on neuroimmunity is also prominent in AD. Altered gut microbiota (e.g., reduced SCFA producers) in AD patients correlates with cerebrospinal fluid tau/Aβ pathology and cognitive decline. Mechanistically, butyrate influences microglial responses to Aβ plaques, exhibiting a complex dual role: facilitating periplaque localization while potentially suppressing phagocytic activity, highlighting its nuanced immunomodulatory properties (53). This body of evidence strongly supports the central hypothesis that gut metabolites act as upstream signals, modulating local and systemic immune status to drive or ameliorate neuroinflammation, ultimately influencing the progression of neuroimmune inflammatory diseases.

In experimental autoimmune encephalomyelitis (EAE) and related models, supplementation with butyrate or propionate has frequently been reported to reduce pro-inflammatory mediators such as IL-17 and TNF-α, restore the Th17/Treg balance, and attenuate demyelination and neurological impairment. These findings, however, should not be taken to mean that short-chain fatty acids (SCFAs) are uniformly protective across all neuroimmune inflammatory diseases.

SCFAs are not functionally interchangeable molecules. Acetate, propionate, and butyrate differ in their microbial sources, intestinal concentrations, absorption kinetics, tissue distribution, and receptor affinities. Butyrate is commonly linked to histone deacetylase (HDAC) inhibition, epithelial energy metabolism, Treg induction, and remyelination (54). Propionate appears to influence peripheral immune-cell metabolism and Treg function (55). Acetate, by contrast, may exert more complex, and in some settings even opposing, immunological effects (56). For example, studies in NMOSD have shown that patients in the acute phase may exhibit gut dysbiosis and acetate deficiency (57). Yet fecal microbiota transplantation from NMOSD patients into germ-free mice, or direct acetate supplementation, has been associated with increased serum IL-6, IL-17A, and IL-23 levels, expansion of Th17 cells, and a reduction in Treg cells (58). These observations suggest that acetate does not necessarily act as an anti-inflammatory metabolite and, under particular immune conditions, may instead amplify Th17-related inflammation.

The biological effects of a given SCFA may also vary according to dose, disease stage, and host immune status (59). At low or physiological concentrations, SCFAs may help preserve intestinal barrier integrity and immune tolerance. In contrast, high concentrations, non-physiological administration, or specific delivery routes may engage different cellular pathways (60). The inflammatory milieu also changes over the course of disease. Acute inflammation is often dominated by pro-inflammatory cytokines, microglial activation, and infiltration of peripheral immune cells, whereas chronic stages may involve tissue repair, remyelination, and immune exhaustion.

Caution is also needed when extrapolating protective effects observed in EAE to patients with MS. EAE remains a valuable model for studying immune mechanisms relevant to MS, but it does not fully reproduce the induction process, disease course, or immune architecture of human MS (61). EAE is typically induced by defined myelin antigens, evolves in a relatively synchronized inflammatory pattern, and is largely driven by T cell-mediated responses. MS, in contrast, is highly heterogeneous and shaped by genetic susceptibility, environmental exposures, prior infections, B-cell responses, chronic neurodegenerative processes, and the long-term effects of disease-modifying therapies (62). Thus, improvements in neurological function after butyrate or propionate supplementation in EAE do not, by themselves, establish equivalent therapeutic efficacy in patients with MS.

Overall, SCFAs are better understood as context-dependent immunometabolic regulators rather than universally protective anti-inflammatory molecules. Their effects depend on the specific SCFA involved, its concentration range, site of action, disease entity, disease stage, and the immune background of the host.

### 6.2 Bile acids

Bile acids, as key gut microbiota-derived metabolites, are increasingly recognized as critical regulators of CNS function, playing complex roles with both protective and damaging potential in various neuroimmune and neurodegenerative diseases. Their dynamic balance is crucial for neurological health (10).

The neuroprotective effects of bile acids are primarily attributed to their anti-inflammatory properties. In neuroimmune diseases, specific bile acid levels correlate with disease activity. For instance, in neuromyelitis optica spectrum disorder (NMOSD), levels of the primary bile acid glycodeoxycholic acid (GUDCA) are higher in patients with low relapse rates and positively correlate with the B-cell chemoattractant CXCL13, suggesting a role in modulating relapse mechanisms (63). The secondary bile acid tauroursodeoxycholic acid (TUDCA) supplementation has demonstrated clear biological effects in clinical studies: in multiple sclerosis (MS) trials, TUDCA treatment safely alters immune cell subset distribution, reducing central memory CD4+ and Th1/17 cells (64). Mechanistically, bile acids exert their effects through receptors such as the Takeda G protein-coupled receptor 5 (TGR5). TGR5 agonists (e.g., ursodeoxycholic acid, tauroursodeoxycholic acid) can effectively suppress neuroinflammation and promote repair in experimental EAE and cerebral ischemia models by inhibiting the NF-κB pathway and activating the Nrf2 signaling cascade, thereby inhibiting excessive activation of microglia and astrocytes and the release of pro-inflammatory factors (17). TUDCA supplementation has also been confirmed to prevent glial cells from polarizing to a neurotoxic phenotype and improves neuropathology in MS animal models (65) (**Figure 1A**).

However, bile acids also possess neurotoxic potential, with effects that are age- and disease-context-specific. In early AD, deoxycholic acid accumulates in the brain and, by binding to the neuronal TGR5 receptor, triggers STAT3 phosphorylation, promotes Aph1 transcription, and increases β-amyloid (Aβ) production, directly impairing cognitive function (18). More profoundly, the dynamic changes of bile acids are closely linked to aging and the gut microbiome. Research has identified an age-related bile acid, tauro-β-muricholic acid (TβMCA), which accumulates in the brains of aged mice and directly induces inflammatory microglia, thereby driving neuroinflammation and cognitive decline. This process is associated with a reduction in gut microbes harboring bile salt hydrolase, and transplantation of gut microbiota from young individuals can reverse these detrimental changes (18) (**Figure 1A**).

In summary, bile acids are dynamic neuromodulators within the gut-brain axis, and their ultimate effects depend on specific species, concentrations, receptor distributions, and microenvironments. They can exert neuroprotective roles through potent anti-inflammatory mechanisms or transform into neurotoxic molecules under specific pathological or aging contexts. Future research needs to further dissect the specific metabolic pathways, cellular targets, and signaling networks of different bile acids within the CNS to precisely regulate their balance in related diseases and provide a basis for developing novel therapeutic strategies.

### 6.3 Other metabolites

Other key ketabolites shaping the BBB and neural Environment. Beyond SCFAs and bile acids (BAs), other gut-derived metabolites play crucial roles in neuro-immune modulation. TMAO, a microbial metabolite of dietary methylamines produced by genera such as Clostridium, Escherichia, and Proteus, has been implicated in neurological processes (66). Indole-3-propionic acid (IPA), generated by *Clostridium sporogenes*, is essential for effective axonal regeneration; its administration enhances regeneration and accelerates sensory recovery after sciatic nerve injury (67). IPA also strengthens blood-brain barrier integrity under both physiological and inflammatory conditions, potentially via activation of endothelial membrane annexin A1 signaling, thereby protecting mice from inflammation-related memory deficits (68). Furthermore, microbial metabolism of amino acids provides an indirect pathway for neuro-modulation: while microbially-derived neurotransmitters (e.g., dopamine, serotonin, GABA) often cannot readily cross the intact blood-brain barrier, their precursor molecules (e.g., tryptophan, tyrosine, L-DOPA) can enter the CNS and modulate local neurotransmitter synthesis and availability, thereby influencing neural circuit activity and neuroendocrine axes closely associated with neuroinflammation (69).

## 7 Neuro-immune interactions

### 7.1 Cellular foundations: from microglia to adaptive immunity

Traditionally viewed as an “immune-privileged” site, the CNS is now recognized as a dynamic interface where immune surveillance is indispensable for maintaining homeostasis and supporting cognitive function (70). This intricate ecosystem extends far beyond passive barrier protection, encompassing a diverse array of resident and border-associated immune cells that engage in constant dialogue with neural cells to orchestrate development, plasticity, and repair.

At the apex of this hierarchy reside microglia, the CNS’s primary resident myeloid cells, which serve as environmental sentinels and homeostatic regulators (71, 72). These cells express a unique molecular signature, including markers such as Siglec-H and P2RY12 (73, 74), allowing them to sense and respond to both neural cues and peripheral immune signals like IL-4 and IFN-γ (75). During development, microglia actively participate in neural circuit refinement by phagocytosing excess neural progenitors and redundant synapses, while also secreting neurotrophic factors. In pathological states, they can transform into “disease-associated microglia” (DAMs), adopting a dualistic role that can either exacerbate or mitigate damage depending on the context.

Astrocytes, the most numerous glial cells, act as crucial intermediaries between the neural and immune compartments (76). They express a broad repertoire of cytokine receptors, enabling them to detect and respond to inflammatory signals in their microenvironment. Beyond their supportive roles, astrocytes also possess compensatory phagocytic capabilities. Their functional state is intricately linked to mitochondrial health, which serves as a key determinant of their regulatory output (77–82). For instance, butyrate has been identified as a significant modulator of astrocytic mitochondrial function, influencing activity through multiple mechanisms, including the upregulation of melatonin pathways, inhibition of HDACs, and optimization of oxidative phosphorylation. While studies in rodent models of autism spectrum disorder (ASD) have demonstrated that propionic acid (PPA), a gut bacterial metabolite, can induce neuroinflammation and reactive astrogliosis (83), a direct causal link between astrocytes and specific human neuroinflammatory diseases such as MS, AD, or Parkinson’s disease (PD) remains to be conclusively established in clinical research.

Microglia and astrocytes collaborate with border-associated macrophages (BAMs)—such as CD206^+^ and LYVE1^+^ macrophages located at the meninges and choroid plexus—to form a comprehensive innate immune defense network (84, 85). BAMs function as professional antigen-presenting cells at these CNS interfaces, capturing CNS-derived antigens and facilitating their presentation to T cells, thereby serving as a critical bridge between central and peripheral immune surveillance (86).

The CNS immune landscape is further enriched by adaptive immune components. T cells, particularly tissue-resident memory T cells (TRM cells) expressing CD69 and CD103, which are found in the meninges, perivascular spaces, and even within brain parenchyma, are increasingly recognized as key modulators of cognitive function and neural plasticity (87, 88). For example, meningeal γδ T cells influence neuronal activity in the prefrontal cortex by secreting IL-17A, thereby regulating anxiety-like behaviors (89). Conversely, regulatory T cells (Tregs) help maintain hippocampal neurogenesis by modulating IFN-γ signaling pathways (90). Different T cell subsets directly communicate with neurons and glia via their signature cytokines (e.g., IL-4, IFN-γ, IL-17), fine-tuning synaptic plasticity, the excitation/inhibition balance, and even memory formation. The role of B cells in the CNS is also more nuanced than previously appreciated. From the CXCL13-CXCR5 axis-dependent recruitment of B-1a cells in the neonatal brain, which support oligodendrocyte development via IgM signaling, to the negative selection of immature B2 cells in the adult meninges to prevent autoimmunity, B cells contribute to both early developmental support and the maintenance of immune tolerance (91). Additionally, innate lymphoid cells (ILCs), particularly ILC2s located in the choroid plexus and meninges, produce cytokines like IL-13 and have been implicated in processes such as inhibitory synapse maturation, the regulation of social behaviors, and the counteraction of cognitive decline associated with brain aging (92).

In summary, the healthy brain harbors a sophisticated, multi-layered, and highly coordinated immune network composed of resident microglia, border-associated macrophages, strategically positioned T and B lymphocytes, and ILCs. This network is not merely a passive defense system but an active, dynamic participant in neural development, circuit remodeling, and the physiological regulation of higher-order functions, providing a crucial cellular and biological foundation for understanding the complex interplay along the “gut-metabolite-immune-brain” axis (**Table 1**).

**Table 1:** Cellular basis of neuroimmunoinflammatory diseases.

Immune cell	Primary location/distribution in the CNS	Key intestinal metabolite regulation mechanisms and impacts	Major neuroimmunoinflammatory diseases	Novel therapeutic strategies/potential targets	References
Microglia	Parenchyma of the CNS	SCFAs: e.g., butyrate, can promote the transition of microglia to an anti-inflammatory phenotype (similar to M2) by inhibiting HDACs, thereby suppressing neuroinflammation. Tryptophan metabolites (e.g., kynurenic acid, indoles): By activating the AhR, they can regulate the metabolic state and function of microglia, inhibit their pro-inflammatory activity, and are crucial for maintaining brain homeostasis.	PD, MS, AD.	Supplementing prebiotics/probiotics to increase SCFA production; small molecule agonists targeting the AhR pathway (e.g., tryptophan derivatives); modulating the microbiota to improve metabolite profiles.	(93–99)
T cells	Dural/leptomeningeal regions, choroid plexus, minority in brain parenchyma	SCFAs: Particularly butyrate and propionate, can enhance the differentiation and function of regulatory T cells (Tregs) through various mechanisms (e.g., promoting epigenetic modifications), thereby suppressing the overactivation of effector T cells. Tryptophan metabolites: Through the AHR pathway, they can promote Treg differentiation while inhibiting the pro-inflammatory activity of Th17 cells, which play a key role in neuroinflammation due to their preference for glycolysis.	CD4^+^ T cells are associated with the pathology of hemorrhagic brain injury; CD8^+^ T cells are associated with neurodegenerative diseases (PD, AD, MS) and various encephalitides.	Using specific probiotic strains to modulate tryptophan metabolism; developing metabolic modulators targeting the glycolysis pathway of Th17 cells or the oxidative phosphorylation pathway of Tregs.	(88, 100–105)
B cells	Dural/leptomeningeal regions, choroid plexus, minority in brain parenchyma	Microbiota-associated molecular patterns and metabolites: The gut microbiota and its metabolites influence systemic and local immunity via the gut-brain axis, potentially involved in regulating B cell activation and autoantibody production, which plays a key role in various autoimmune neurological diseases.	MS, NMOSD, Anti-NMDA receptor encephalitis, AE, etc.	Targeting specific pathogenic B cell clones; using probiotics to modulate overall immune homeostasis to indirectly influence B cell responses.	(91, 106–108)
BAMs	CNS borders: meninges, choroid plexus, perivascular spaces	Circulating gut metabolites: Metabolites such as SCFAs can reach CNS border regions via the bloodstream, modulating the phenotype and function of these macrophages, affecting their ability to clear waste, maintain blood-brain barrier integrity, and regulate neuroinflammation.	Play roles in PD, AD and MS.	Developing metabolite-derived drugs capable of penetrating or acting on the immune environment outside the blood-brain barrier.	(86, 109–114)
Innate lymphoid cells	Dural/leptomeningeal regions, choroid plexus, minority in brain parenchyma	Metabolic reprogramming and microbiota: The gut microbiota directly influences the development and function of ILCs (especially ILC3). Upon activation, ILC3 undergo significant metabolic reprogramming (enhanced glycolysis, fatty acid synthesis) and produce IL-22, which is crucial for maintaining the intestinal barrier and potentially CNS immune homeostasis. Microbiota dysbiosis can lead to ILC dysfunction and contribute to neuroinflammation.	Present in the meninges under homeostasis; migrate to the brain parenchyma during stroke and MS.	Modulating the metabolic state and function of ILCs through dietary or microbiota interventions, such as supplementing ω-3 polyunsaturated fatty acids.	(92, 115)

SCFAs, Short-chain fatty acids. HDACs, histone deacetylases. AhR, aryl hydrocarbon receptor. CNS, central nervous system. NMOSD, neuromyelitis optica spectrum disorders. MS, Multiple sclerosis. AE, autoimmune epilepsy. PD, Parkinson’s disease. AD, Alzheimer’s disease. BAMs, Border-associated macrophages.

### 7.2 Immunity in neuroinflammatory diseases: from homeostatic guardian to disease driver

Within the CNS, the immune response assumes a critical and paradoxical dual role: it serves as a cornerstone for maintaining tissue homeostasis, supporting neurodevelopment and repair, while simultaneously acting as a core driver of neuroinflammation and disease pathology. This seemingly contradictory duality is primarily orchestrated through complex, dynamic interactions between resident innate immune cells—such as microglia and astrocytes—and peripherally recruited or brain-resident adaptive immune cells (T cells, B cells), collectively forming the pathophysiological basis of neuroimmune-inflammatory disorders (116).

Under physiological conditions, microglia and astrocytes, as the central executors of CNS innate immunity, actively participate in synaptic pruning, provide neuronal trophic support, and maintain microenvironmental homeostasis. However, upon pathological stimulation, both can be rapidly activated, establishing a pro-inflammatory positive feedback loop. Microglia, through the release of factors like IL-1α, TNF-α, and complement C1q, activate the NF-κB pathway in astrocytes, inducing their transformation into a reactive phenotype. In turn, reactive astrocytes feedback with TNF-α and IL-6, further exacerbating the inflammatory state of microglia (117–119). This aberrant glial crosstalk leads to a loss of neuronal support functions and direct neurotoxicity, including IL-1β-mediated synaptic dysfunction and TNF-α-activated caspase cascades (120, 121). In AD, such an inflammatory milieu not only impairs the clearance of β-amyloid (Aβ) but may also promote its production, creating a vicious cycle (122). Furthermore, activated glial cells generate reactive oxygen species (ROS), nitric oxide (NO), and chemokines (e.g., CCL5), causing direct oxidative damage and recruiting peripheral T and B cell infiltration, thereby amplifying the inflammatory cascade (123). Notably, immune responses exhibit high context-dependency and temporal heterogeneity. For instance, in acute injuries like ischemic stroke, microglia and astrocytes may initially exhibit anti-inflammatory, phagocytic, and reparative phenotypes (expressing Arg1, IL-10, etc.), aiding in debris clearance and damage limitation. However, they may shift towards a pro-inflammatory phenotype in later stages, exacerbating tissue injury. Concurrently, astrocytes play a crucial protective role in demarcating injury areas, mitigating ROS accumulation, and promoting vascular repair (124, 125). Border-associated macrophages (BAMs), acting as perivascular innate immune sentinels, are vital in regulating blood-brain barrier permeability, clearing Aβ (in AD models), and initiating T cell responses as antigen-presenting cells (e.g., in Parkinson’s disease α-synuclein models) (113, 126). Innate lymphoid cells (ILCs) also participate in regulation; for example, meningeal ILC2s can promote tissue repair by secreting neurotrophic factors like calcitonin gene-related peptide (α-CGRP) (127).

The infiltration and functional modulation of adaptive immune cells, particularly T lymphocytes, profoundly influence the course and outcome of neuroinflammation. In autoimmune diseases like multiple sclerosis (MS), the recognition of myelin antigens by autoreactive CD4^+^ T cells initiates CNS inflammation and demyelination, a process exacerbated by B cells through antigen presentation, T cell help, and autoantibody production (128). However, T cell function is highly heterogeneous. Following ischemic stroke or traumatic brain injury, infiltrating CD4^+^ T cells can differentiate into various effector subsets. Regulatory T cells (Tregs) accumulate at injury sites and exert potent anti-inflammatory and tissue-reparative functions by producing molecules like IL-10, TGF-β, and amphiregulin, for example, by inhibiting neurotoxic astrogliosis and reducing pro-inflammatory cytokine production, thereby promoting neurological recovery (129, 130). Conversely, cytotoxic CD8^+^ T cells exacerbate neuroinflammation and damage by directly inducing neuronal death via the release of perforin and granzymes (131). Adaptive immune infiltration is now a recognized feature of neurodegenerative diseases. In tauopathy models of AD, intracerebral infiltration of CD8^+^ effector T cells has been shown to directly drive tau-mediated neurodegeneration (132). Clinical studies have also observed lymphocyte infiltration in the brains of AD and Parkinson’s disease (PD) patients, suggesting its role as a common pathological component and a potential therapeutic target (133, 134). More intriguingly, adaptive immunity even plays a role in neurodevelopmental disorders such as autism spectrum disorder (ASD). Maternal immune activation (MIA) models reveal that the gut microbiota can influence maternal immune status, leading to meningeal γδ T cell production of IL-17A. This cytokine, by acting on neuronal IL-17A receptors or activating border-associated macrophages to produce ROS, ultimately induces anxiety-like behavior and cognitive deficits in offspring (89, 135, 136). Interestingly, IL-17A demonstrates a paradoxical capacity to restore neurotypical behavior in certain contexts, highlighting the extreme complexity of neuroimmune signaling networks (137). Thus, from autoimmune attack and post-injury repair to neurodegeneration and even developmental abnormalities, adaptive immune cells, through their heterogeneous functional manifestations, are deeply embedded within the pathophysiological network of neuroimmune inflammatory diseases.

Given the central driving role of immune cells in neuroinflammatory diseases, targeted modulation of immune dynamics has become a key therapeutic strategy. In neurotrauma and vascular diseases, such as post-stroke application of fingolimod or anakinra, or through Treg expansion, microglial activity can be effectively regulated to mitigate neuroinflammation (138). For neurodegenerative diseases, immunotherapies targeting aberrant protein aggregates (e.g., anti-Aβ antibodies aducanumab/lecanemab, vaccines targeting α-synuclein aggregates) and inhibitors targeting neuroimmune pathways (e.g., masitinib inhibiting microglia/mast cells, monoclonal antibodies blocking the PD-1/PD-L1 pathway) represent the current frontier of clinical translation (139, 140). For neurodevelopmental disorders (e.g., ASD, pediatric epilepsy) and psychiatric disorders (e.g., depression, anxiety), immunomodulation also shows promise, exemplified by Treg expansion therapies, cytokine antagonists (tocilizumab, anakinra), and antibody-targeted treatments (natalizumab) (141). These strategies collectively point towards a central goal: to restore immune homeostasis by precisely targeting and modulating dysregulated neuroimmune pathways, thereby intervening in or even reversing disease progression.

## 8 Novel therapeutic strategies for neuroimmune inflammatory diseases: targeting gut-derived metabolites

In recent years, targeting gut-derived metabolites to modulate the “gut-brain axis” has emerged as a promising frontier in treating neuroimmune inflammatory diseases. The gut microbiota, through its complex metabolic activities, produces a variety of bioactive molecules, including SCFAs, secondary bile acids (SBAs), tryptophan derivatives, and TMAO. These metabolites can cross the blood-brain barrier and directly or indirectly regulate immune responses and neural functions in the CNS. The core therapeutic strategy involves restoring the homeostatic balance of the host’s immune-neural network by either directly supplementing key metabolites or indirectly reshaping the microbial metabolic network.

### 8.1 Direct supplementation strategy: precise delivery of key metabolites

**Figure 2:**
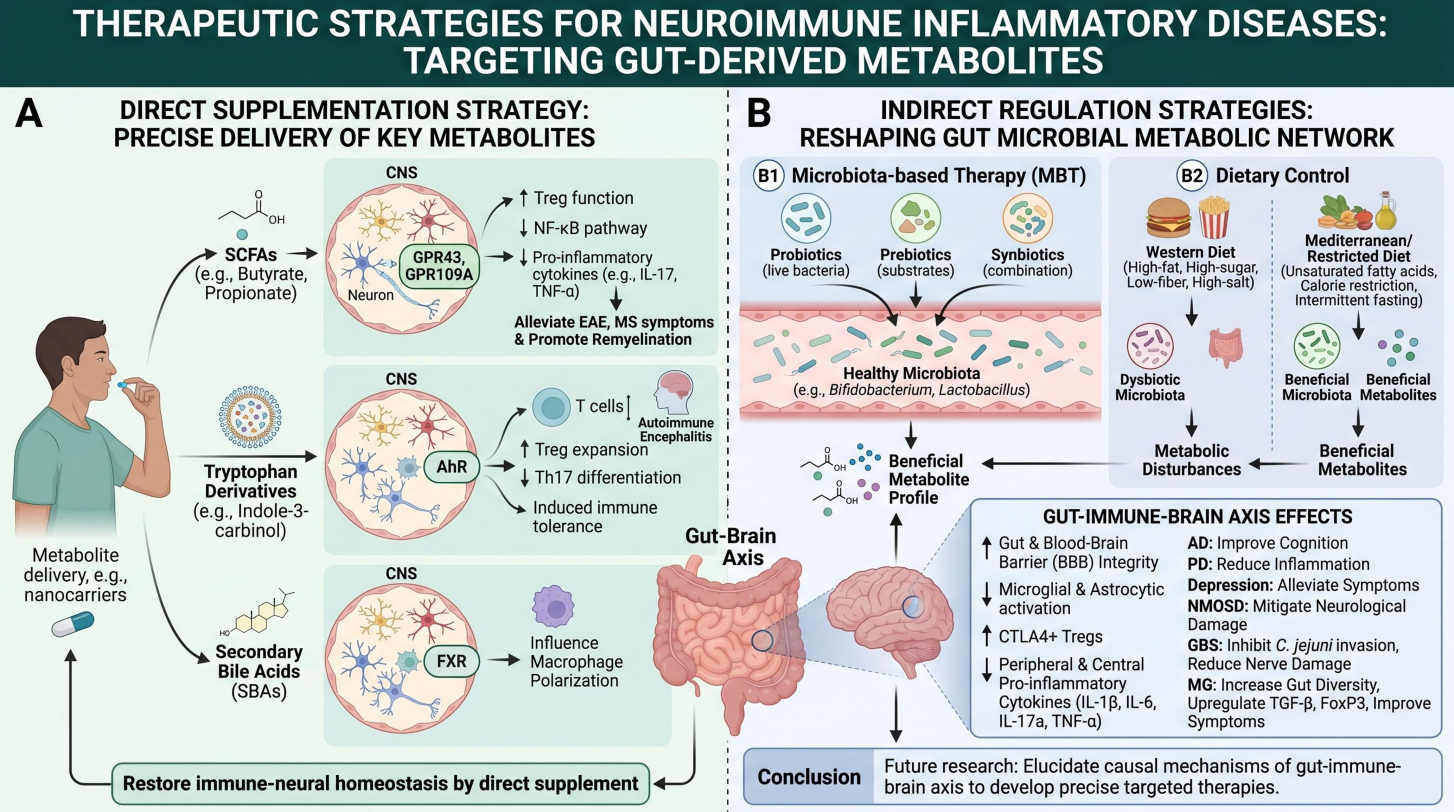
Therapeutic strategies targeting gut-derived metabolites to modulate the gut–immune–brain axis in neuroimmune inflammatory diseases. **(A)** Direct supplementation strategies involve the targeted delivery of key gut-derived metabolites, including SCFAs, tryptophan derivatives, and SBAs, via oral formulations or nanocarrier-based systems. SCFAs such as butyrate and propionate may activate GPR43 and GPR109A, enhance Treg function, inhibit NF-κB signaling, reduce IL-17 and TNF-α production, alleviate EAE and MS-related symptoms, and promote remyelination. Tryptophan derivatives may regulate T-cell responses through AhR signaling by promoting Treg expansion, suppressing Th17 differentiation, and inducing immune tolerance. SBAs may signal through receptors such as FXR to modulate macrophage polarization and neuroinflammatory responses. **(B)** Indirect regulation strategies aim to reshape the gut microbial metabolic network through microbiota-based therapies and dietary interventions. Probiotics, prebiotics, and synbiotics may restore beneficial bacteria, including *Bifidobacterium* and *Lactobacillus*, and increase protective metabolite production. In parallel, healthy dietary patterns such as Mediterranean diets, calorie restriction, and intermittent fasting may improve microbial and metabolic homeostasis, whereas Western diets are associated with dysbiosis and metabolic disruption. By optimizing gut-derived metabolite profiles, these approaches may enhance gut and blood–brain barrier integrity, suppress glial activation and pro-inflammatory cytokine production, increase CTLA4^+^ Tregs, and improve outcomes in multiple neuroimmune and neurological disorders. Further studies are required to define the causal mechanisms of the gut–immune–brain axis and support precision therapeutic development.

The direct supplementation strategy aims to precisely deliver metabolites with defined immunomodulatory functions. For example, SCFAs, such as butyrate and propionate, which are primary products of dietary fiber fermentation, exert potent anti-inflammatory effects by activating G protein-coupled receptors (e.g., GPR43, GPR109A). In experimental EAE and MS models, supplementation with butyrate or propionate significantly alleviates neurological symptoms, a mechanism involving the inhibition of the NF-κB pathway, reduction in the production of pro-inflammatory cytokines (e.g., IL-17, TNF-α), and enhancement of regulatory T cell (Treg) function. Preclinical studies also suggest that butyrate has the potential to promote remyelination (142). In patients with NMOSD, fecal SCFA levels (e.g., acetate, butyrate) are reduced and negatively correlate with disease severity (143), implying that their supplementation may have therapeutic value. However, this requires delivery systems, such as nanocarriers, to overcome their low bioavailability. Another important class of metabolites is tryptophan derivatives, such as indole-3-carbinol. These activate the aryl hydrocarbon receptor (AhR), promoting Treg expansion and inhibiting Th17 cell differentiation, thereby inducing immune tolerance. Clinical trials have shown that AhR agonists combined with conventional immunotherapy can improve neurological function in patients with autoimmune encephalitis, but careful dose optimization is necessary to avoid side effects (**Figure 2A**).

### 8.2 Indirect regulation strategies: reshaping the gut microbial metabolic network

Indirect regulation strategies focus on modulating the host environment—either through dietary intervention or direct manipulation of the microbial community—to reshape a beneficial metabolite profile (144), thereby exerting therapeutic effects on neuroinflammatory diseases. Microbiota-based therapies (MBT) and dietary control have emerged as prominent approaches in this context (**Figure 2B**).

#### 8.2.1 MBT

MBT involves the administration of live microorganisms (probiotics), substrates that support their growth (prebiotics), or their bioactive metabolites to restore gut ecological balance and function, which in turn indirectly modulates neuroimmune responses (144). The core mechanisms include enhancing the integrity of the gut and blood-brain barriers and reshaping the local immune environment in both the gut and the brain. For instance, in AD models, a probiotic mixture containing *Lactobacillus paracasei* and *Lactobacillus rhamnosus* was shown to improve cognition by reducing activated microglia and lowering levels of systemic and brain IL-6 and TNF-α (145). In Parkinson’s disease models, either a synbiotic (e.g., *Lactobacillus paracasei* DG with inulin) or probiotic combinations significantly reduced plasma levels of IL-6 and TNF-α in patients (146). For depression models, specific synbiotics and probiotic mixtures alleviated behavioral symptoms by promoting the expansion of gut CTLA4+ regulatory T cells (Tregs) and suppressing Th17 cells along with peripheral and central pro-inflammatory cytokines (IL-1β, IL-17a, TNF-α) (147). Transplanting fecal microbiota from NMOSD patients into germ-free mice revealed the colonization of various *Clostridium* species, which were found to have dual roles: promoting Treg proliferation (anti-inflammatory) and Th17 proliferation (pro-inflammatory) (142, 148). Collectively, these findings highlight the pivotal role of gut metabolites, particularly SCFAs, in intervening in neuroinflammatory disease pathways by reshaping host immunity. Future research needs to delve deeper into the specific molecular mechanisms by which certain microbes or their metabolites regulate the causal chain of the “gut-immune-brain” axis to drive the development of more precise targeted therapies.

Certain microbial metabolites, such as SCFAs and vitamin D, not only provide nutritional benefits to the host but also participate in the regulation of the gut microbiome and immune system, helping to maintain gut homeostasis (149, 150). Studies have shown that probiotics can inhibit Th1/Th17 polarization in EAE mice and promote the production of IL-10 and Tregs, exerting neuroprotective effects (150). Commercial probiotics composed of different strains of *Lactobacillus*, *Bifidobacterium*, and *Streptococcus* have been found to dose-dependently mitigate dendritic cell differentiation and inflammatory infiltration, protecting neural function in EAE mice (151). Small-scale clinical studies have also demonstrated that probiotics can improve intestinal micro-ecological diversity in MS patients, reduce lesion numbers, and decrease clinical relapses (152). In animal models of GBS, probiotics can inhibit the colonization and invasion of *Campylobacter jejuni* in the small intestine epithelial cells (153). Clinical research has found that a GBS patient treated with probiotics showed markedly improved peripheral nerve damage (154). *Bifidobacterium* and *Lactobacillus* can increase the diversity of the gut microbiota in myasthenia gravis (MG) rat models and improve clinical symptom scores, with symptom improvement associated with decreased serum anti-AChR antibody levels and increased expression of TGF-β and FoxP3 in lymph nodes and spleen, as well as upregulated TLR2 expression in bone marrow-derived dendritic cells (155). Therefore, probiotics are of great benefit in mitigating neurological functional impairment and improving clinical symptoms in neuroimmune diseases.

#### 8.2.2 Dietary control

Dietary patterns characterized by high fat, high sugar, and low fiber (typical Western diets) are prone to causing gut microbial dysbiosis, reducing gut immunity, and exacerbating inflammatory responses and neural functional damage in EAE mice (156). In contrast, a “Mediterranean diet” rich in unsaturated fatty acids can reduce oxidative stress, alleviate myelin damage, and improve EAE symptoms (157). High-salt diets, which mimic Western dietary patterns, promote the infiltration of Th17 cells into the CNS and reduce levels of lactobacilli, which have protective effects on the gut barrier, thereby exacerbating neural functional damage in EAE mice (158, 159). Methods such as “calorie restriction” (involving very low-calorie and low-protein diets) (159) and “intermittent fasting” (feeding mice every other day) (160) have been shown to improve neural functional damage in EAE mice and increase the abundance of bacteria from the families Bacteroidaceae and Lactobacillaceae, with mechanisms potentially related to complex metabolic, hormonal, and endocrine responses (161). Diets rich in vitamin D, which can promote IL-10 secretion, have long been known to improve clinical symptoms in MS patients. Some studies have found that moderate alcohol consumption can reduce the proliferation and differentiation of microglia in EAE mice, thereby alleviating neural functional damage, with a more pronounced effect observed in male mice (162). Therefore, dietary adjustments are beneficial for MS patients in reducing inflammation and improving symptoms. However, research on the relationship between diet and other neuroautoimmune diseases is still limited.

Dysbiosis-induced metabolic disturbances can trigger microglial overactivation, astrocyte proliferation, and peripheral immune dysregulation, thereby exacerbating neuroinflammation and injury. Beyond their canonical roles via GPRs, SCFAs exert immunomodulatory effects by inhibiting HDACs to promote regulatory T cell (Treg) differentiation. Secondary bile acids influence macrophage polarization through the farnesoid X receptor (FXR), while tryptophan metabolites modulate the Th17/Treg balance via the AhR pathway. Emerging evidence further indicates that targeting gut metabolites contributes to the restoration of blood-brain barrier integrity and reduces the leakage of inflammatory mediators into the CNS. In summary, current evidence underscores the pivotal role of gut-derived metabolites in reshaping host immunity and their therapeutic potential in intervening in neuroinflammatory disease pathways. Future research needs to delve into the specific molecular mechanisms by which distinct microbes or their metabolites regulate the causal chain of the “gut-immune-brain” axis to drive the development of more precise targeted therapies.

## 9 Conclusion

Gut-derived metabolites provide a dynamic biochemical link between the intestinal ecosystem and neuro-immune regulation. Metabolites such as short-chain fatty acids, bile acids, and tryptophan-derived compounds can influence microglial and astrocytic activation, shape the balance between regulatory and effector immune responses, and contribute to the maintenance or disruption of barrier integrity. These findings have broadened our understanding of neuro-immune inflammatory diseases, suggesting that disease activity is not determined solely by CNS-intrinsic mechanisms, but also by systemic metabolic and microbial signals.

Therapeutically, this field is moving in two converging directions. One approach targets downstream immune pathways directly, using strategies such as cytokine blockade, kinase inhibition (163), Treg-based therapies, or antibodies directed against disease-associated protein aggregates. The other seeks to modify upstream microbial and metabolic inputs through diet, probiotics, prebiotics, synbiotics, or selected metabolite supplementation (164). Preclinical studies have provided encouraging evidence that such interventions can attenuate neuroinflammation (165), improve barrier function, and restore immune balance. However, these findings should be interpreted carefully. Much of the current evidence still comes from animal models, while well-powered human studies remain limited. Differences in diet, sample collection, metabolite measurement, and study design also make results difficult to compare across cohorts.

A further challenge is biological interpretation. Fecal metabolite levels do not necessarily reflect circulating concentrations, CNS exposure, or tissue-specific bioactivity. Gut metabolites rarely act in isolation; rather, they operate within interconnected networks in which SCFAs, bile acids, tryptophan metabolites, and other microbial products may reinforce, counteract, or reshape one another’s effects. Future studies therefore need to move beyond simple abundance-based associations and define causal, cell-specific, and disease-stage-specific mechanisms (**Figure 2**).

Overall, gut metabolite biology offers a compelling framework for understanding neuro-immune inflammation and for developing new therapeutic strategies. Its clinical translation, however, will depend on rigorous longitudinal human cohorts, standardized metabolomic approaches, and well-controlled interventional trials that integrate microbiome, metabolome, immune, and clinical endpoints. With this level of evidence, microbiota- and metabolite-targeted interventions may eventually complement conventional immunomodulatory therapies and enable more precise restoration of neuro-immune homeostasis.
